# Human ACE2 protein is a molecular switch controlling the mode of SARS-CoV-2 transmission

**DOI:** 10.1186/s12929-023-00980-w

**Published:** 2023-10-12

**Authors:** Chao-Fu Yang, Chun-Che Liao, Hung-Wei Hsu, Jian-Jong Liang, Chih-Shin Chang, Hui-Ying Ko, Rue-Hsin Chang, Wei-Chun Tang, Ming-Hao Chang, I-Hsuan Wang, Yi-Ling Lin

**Affiliations:** 1 Institute of Biomedical Sciences, Academia Sinica, Taipei, 11529 Taiwan; 2 Biomedical Translation Research Center, Academia Sinica, Taipei, 11529 Taiwan; 3 Research Center for Applied Sciences, Academia Sinica, Taipei, 11529 Taiwan

**Keywords:** SARS-CoV-2, COVID-19, ACE2, Cell-free transmission, Cell-to-cell transmission

## Abstract

**Background:**

Human angiotensin-converting enzyme 2 (hACE2) is the receptor mediating severe acute respiratory syndrome coronavirus 2 (SARS-CoV-2) infection. hACE2 expression is low in the lungs and is upregulated after SARS-CoV-2 infection. How such a hACE2-limited pulmonary environment supports efficient virus transmission and how dynamic hACE2 expression affects SARS-CoV-2 infection are unclear.

**Methods:**

We generated stable cell lines with different expression levels of hACE2 to evaluate how the hACE2 expression level can affect SARS-CoV-2 transmission.

**Results:**

We demonstrated that the hACE2 expression level controls the mode of SARS-CoV-2 transmission. The hACE2-limited cells have an advantage for SARS-CoV-2 shedding, which leads to cell-free transmission. By contrast, enhanced hACE2 expression facilitates the SARS-CoV-2 cell-to-cell transmission. Furthermore, this cell-to-cell transmission is likely facilitated by hACE2-containing vesicles, which accommodate numerous SARS-CoV-2 virions and transport them to neighboring cells through intercellular extensions.

**Conclusions:**

This hACE2-mediated switch between cell-free and cell-to-cell transmission routes provides SARS-CoV-2 with advantages for either viral spread or evasion of humoral immunity, thereby contributing to the COVID-19 pandemic and pathogenesis.

**Supplementary Information:**

The online version contains supplementary material available at 10.1186/s12929-023-00980-w.

## Introduction

Severe acute respiratory syndrome coronavirus 2 (SARS-CoV-2) causes coronavirus disease 2019 (COVID-19); in only a few months, COVID-19 epidemic in Wuhan, China, became a global pandemic. Studying the transmission mechanism of SARS-CoV-2 will provide insights into the reasons for its high transmissibility, which can be used to formulate strategies to suppress its spread. Viruses can spread among cells via two routes: cell-free virion and cell-to-cell transmission, and each route has its advantages and disadvantages [1, 2]. The advantage of cell-free transmission, a major route for the interhost transmission, is that it facilitates the infection of remote targets. However, cell-free virions are vulnerable to physical or immune attacks. Enveloped viruses can spread directly from one cell to another without being released into the extracellular environment, which is known as cell-to-cell transmission; they can avoid immune attacks from neutralizing antibody (NAb) [1, 2].

Cell-free virion infection is the standard method used to conduct experiments in SARS-CoV-2-related studies. Cell-free viruses shed into the extracellular space are used not only to evaluate the effectiveness of various anti-SARS-CoV-2 drugs in vitro, but also to determine the infectious capacity of patients with COVID-19 [3]. The cell-to-cell transmission of SARS-CoV-2 has also been demonstrated in different cells [4–6], and this route can facilitate the infection of SARS-CoV-2-nonpermissive cells [6]. SARS-CoV-2 infection induces the formation of filopodia [7], and SARS-CoV-2 particles have been observed on filopodia and tunneling nanotubes [6, 7], indicating the possible role of intercellular extensions in SARS-CoV-2 cell-to-cell transmission. However, the mechanisms underlying the cell-free and cell-to-cell transmission of SARS-CoV-2 and the factors determining the route remain unclear.

Angiotensin-converting enzyme 2 (ACE2) is the major SARS-CoV-2 receptor, and it interacts with the receptor binding domain (RBD) of viral spike (S) glycoproteins [8]. Although the lung is the target of SARS-CoV-2, human ACE2 (hACE2) expression is very low in the lung and is limited to type II alveolar epithelial cells [9–11]. How this hACE2-limited environment can support SARS-CoV-2 infection remains unclear. *ACE2* is an interferon-stimulated gene (ISG), and its expression is upregulated after virus infection in humans [10]. In this study, we investigated whether and how dynamic hACE2 expression contributes to SARS-CoV-2 infection, especially virus transmission. To address these questions, we generated stable cell lines with different expression levels of hACE2 to evaluate how the hACE2 expression level can affect SARS-CoV-2 transmission.

## Methods

### Cells, viruses, and chemicals

Human lung epithelial A549 cells (CCL-185, ATCC) were maintained in F-12 medium (Thermo Fisher) supplemented with 10% fetal bovine serum. A549 cells stably expressing hACE2 (hACE2-A549 cells) were kindly gifted by Dr. Chia-Yi Yu (National Institute of Infectious Diseases and Vaccinology, National Health Research Institutes, Taiwan). African green monkey kidney Vero E6 cells (CRL-1586, ATCC) were maintained in Eagle’s minimum essential medium (Thermo Fisher) supplemented with 10% fetal bovine serum.

The expression of exogenous genes was achieved using a lentivirus system. hACE2-A549 cells were transduced with RFP-expressing lentiviruses from RNA Technology Platform and Gene Manipulation Core (RNAi Core, Taiwan) to generate hACE2-RFP-A549 cells. A549 cells were transduced with mCherry-expressing lentiviruses (RNAi Core) to generate mCherry-A549 cells. To generate hACE2-A549 clones, single cell was sorted from the population of hACE2-A549 cells by using a FACSJazz-6 color cell sorter (BD Biosciences). These clones were cultured in 96-well plates for 1 week and then transferred to 6-well plates for 4 days to obtain sufficient cells for further experiments. The hACE2 expression level of each hACE2-A549 clone was analyzed using IFA and then confirmed through Western blotting with anti-ACE2 antibody.

SARS-CoV-2 (hCoV-19/Taiwan/4/2020, GISAID accession ID: EPI_ISL_411927) isolated from a patient with COVID-19 was obtained from the Taiwan Centers of Disease Control. The virus was amplified in Vero E6 cells and the virus titer was determined using a tissue culture infective dose assay.

We used the following primary antibodies: anti-tubulin rabbit monoclonal antibody (mAb) (#2128, Cell Signaling), anti-ACE2 rabbit mAb (GTX01160), anti-SARS-CoV-2 S protein mouse mAb (GTX632604), anti-SARS-CoV-2 nucleocapsid protein (N) mouse mAb (GTX632269), anti-SARS-CoV-2 nonstructural protein 3 (NSP3) rabbit polyclonal antibody (GTX135589) (GTX all from GeneTex), and anti-SARS-CoV-2 S protein humanized monoclonal antibody (hmAb; kindly gifted by Dr. An-Suei Yang, Genomics Research Center, Academia Sinica, Taiwan). Secondary antibodies included goat Alexa Fluor 488-conjugated anti-mouse, Alexa Fluor 488-conjugated anti-human, and Alexa Fluor 568-conjugated anti-rabbit IgG antibodies (Thermo Fisher).

Neutralizing hmAbs against the RBD of SARS-CoV-2 S protein were kindly gifted by Dr. Han-Chung Wu [12] (Institute of Cellular and Organism Biology, Academia Sinica, Taiwan). The control hmAb was kindly gifted by Dr. Kuo-I Lin (Genomics Research Center, Academia Sinica, Taiwan).

### Virus transmission assays

For cell-free infection, hACE2-A549 cells were infected with SARS-CoV-2, which was premixed with antibodies or plasma at 37 °C for 1 h, at a multiplicity of infection (MOI) of 0.2. At 1 h post infection (hpi), additional antibody- or plasma-containing medium was added. The supernatant was harvested for the virus infectivity assay, and cells were fixed for IFA with anti-NSP3 antibody and high-content image analysis at 24 hpi.

For the coculture system, hACE2-A549 cells were infected with SARS-CoV-2 at an MOI of 0.2 for 24 h for obtaining the virus donor cells. The virus donor cells were trypsinized and cocultured with virus recipient cells (hACE2-RFP-A549 cells) at a 1:1 ratio in antibody- or plasma-containing medium. The supernatant was harvested for the virus infectivity assay, and at 24 h after coculture, the cells were fixed and subjected to IFA with anti-NSP3 antibody and high-content image analysis.

For temporal viral spread analysis, hACE2-A549 clones at approximately 90% confluence seeded in 96-well plates were infected with SARS-CoV-2 at an MOI of 0.01. At 1 hpi, the medium was replaced with antibody-containing medium. At 24, 48, and 72 hpi, the supernatant was harvested for the virus infectivity assay, and the cells were fixed and subjected to IFA with anti-NSP3 antibody and high-content image analysis.

For the virus infectivity assay, hACE2-A549 cells with approximately 50% confluence were seeded in 96-well plates and then incubated with the supernatant harvested from the virus transmission assay. At 24 h after culture, the cells were fixed and subjected to IFA with anti-NSP3 antibody.

For the viral shedding assay, hACE2-A549 clones were adsorbed with SARS-CoV-2 at an MOI of 0.2 for 1 h. After the removal of the viral inoculant, the cells were washed twice with phosphate-buffered saline (PBS) and incubated in fresh medium. At 24 hpi, the supernatant was harvested and viral RNA was extracted using the RNeasy Mini Kit (Qiagen) and was quantified using real-time reverse transcription–polymerase chain reaction (RT-PCR) with primers targeting the E gene of SARS-CoV-2, as described previously [13]. The viral RNA copy number was determined using a real-time RT-PCR standard generated from a synthetic oligonucleotide fragment of E gene (Genomics BioSci and Tech).

All experiments involving SARS-CoV-2 were conducted in a biosafety level 3 laboratory in accordance with the guidelines established by the Biosafety Level 3 Facility of the Institute of Biomedical Sciences (IBMS), Academia Sinica, Taipei, Taiwan.

### Immunofluorescence assay (IFA) and fluorescence staining

Cells were seeded in 96-well plates or on coverslips for 24 h. After infection, the cells were fixed with 4% paraformaldehyde at room temperature for 20 min, permeated with 0.5% Triton X-100 buffer for 2 min, and blocked with 3% BSA for 30 min. The cells were stained with primary antibodies at room temperature for 1 h, washed three times with PBS, and incubated with secondary antibodies, DAPI (Sigma) for nuclear staining, or Alexa Fluor 647 phalloidin (Thermo Fisher) for F-actin staining at room temperature for 1 h. Viral RNA (vRNA) was stained using the RNAscope Multiplex Fluorescent V2 Assay kit (ACD) with a SARS-CoV-2-specific probe (NC_045512.2, ACD).

For high-content image analysis, immunofluorescence images were acquired using the ImageXpress Micro XLS Widefield high-Content Analysis System (Molecular Devices), and the virus infection rate was measured using MetaXpress Software (Molecular Devices), as described previously [14]. To obtain super-resolution immunofluorescent images, images were acquired using a Zeiss LSM880 confocal microscope equipped with Airyscan [15].

### Correlative light and electron microscopy (CLEM)

First, 5 × 10^5^ hACE2-A549 cells were infected with SARS-CoV-2 at an MOI of 0.2 for 24 h and fixed with 4% paraformaldehyde and 0.2% glutaraldehyde in PBS at room temperature for 1 h. The fixed cells were washed twice with PBS for 5 min before immunofluorescence staining as described above. The immunofluorescence images were acquired using a Zeiss LSM700 microscope. Next, the same sample was incubated with 0.1% OsO4 in 0.1 M PBS for postfixation, dehydrated, and embedded in Spurr’s resin for further transmission electron microscopy (TEM) examination. Ultrathin Sects. (100 nm) were examined using Tecnai G2 Spirit TWIN TEM (Thermo Fisher). Subsequently, for precise interpretation of CLEM data, both immunofluorescence and TEM images were aligned using the ec-CLEM plugin on the Icy platform.

### Statistical analysis

Data are presented as mean ± SD from three independent samples. Statistical significance was set at p < 0.05, and the data were analyzed using two-tailed Student’s *t* test.

## Results

### Cell-free and cell-to-cell transmission of SARS-CoV-2 in human lung cells

**Fig. 1:**
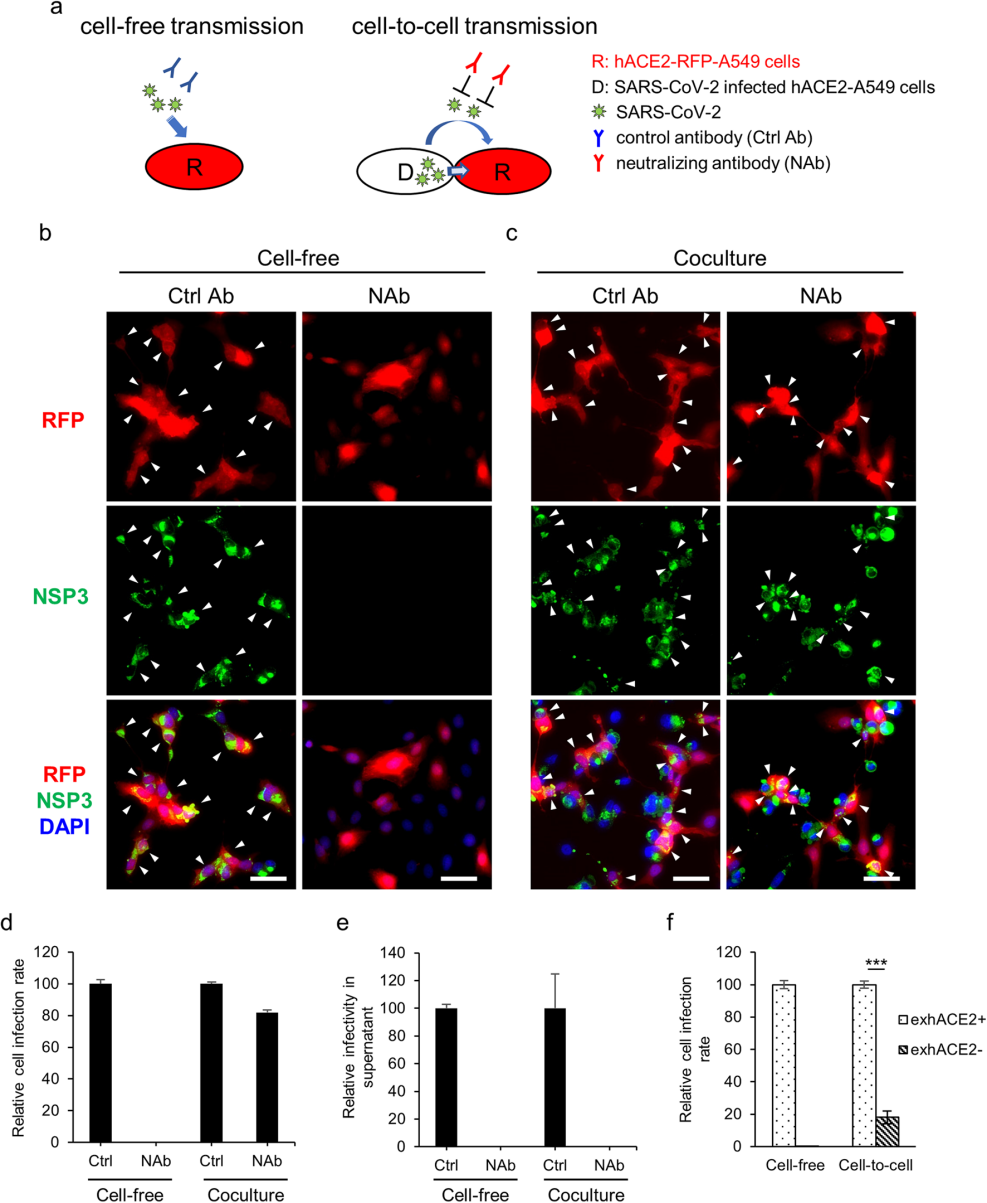
Cell-free and cell-to-cell transmission of SARS-CoV-2 in lung cells. **a** Schematic viral transmission assay. **b–d** Cell-free infection: SARS-CoV-2 was preincubated with neutralizing antibody (NAb) or control antibody (Ctrl Ab) and then used to infect hACE2-RFP-A549 cells (R) for 24 h. Coculture system: SARS-CoV-2-infected hACE2-A549 cells (MOI = 0.2, 24 hpi) were used as virus donor cells (D) and cocultured with hACE2-RFP-A549 cells with NAb or Ctrl Ab for 24 h. NSP3 expression was analyzed using IFA (**b** and** c**) and the infection of R cells was quantified with a high-content image analysis system (**d**). The supernatant was harvested for the virus infectivity assay (**e**). **f** mCherry-A549 cells (without exogenous hACE2 expression, exhACE2-) and hACE2-RFP-A549 cells (with exogenous hACE2 expression, exhACE2 +) were used as R cells for cell-free infection and cell-to-cell transmission assay. The infection of R cells was quantified with a high-content image analysis system at 24 hpi. Arrowhead, SARS-CoV-2-infected R cells; scale bar, 50 μm; Ctrl Ab groups (**d** and **e**) and exhACE2 + groups (**f**) were defined as 100%; all data indicated means with standard deviation (SD) (n = 3) of each group; ***p < 0.001, determined using two-tailed unpaired Student’s t test

The human lung epithelial A549 cell line can serve as a model for type II alveolar epithelial cells with limited amount of hACE2 expression. Therefore, A549 cell with exogenous expression of hACE2 is commonly employed in the study of SARS-CoV-2 infection [7, 16–19]. In this study, we used hACE2-expressing A549 (hACE2-A549) cells in viral transmission assays for evaluating the cell-free and cell-to-cell transmission of SARS-CoV-2 (Fig. 1a).

In cell-free infection, hACE2-A549 cells with red fluorescent protein (RFP, hACE2-RFP-A549) were readily infected by SARS-CoV-2 in the presence of control antibody, as revealed by an IFA with NSP3 (Fig. 1b, Ctrl Ab) and as quantified using high-content image analysis (Fig. 1d). This infection could be completely blocked by premixing SARS-CoV-2 with an NAb against the RBD of S protein [12] (Fig. 1b and d, NAb). Similar results were also obtained using the convalescent plasma from patients with COVID-19 instead of the NAb (Additional file 1: Fig. S1). The abolishment of remaining viral infectivity in the culture supernatants by NAb indicated that it was effective for blocking SARS-CoV-2 infection (Fig. 1e), and that cell-free transmission was sensitive to NAb neutralization.

Distinct results were noted for the coculture system, in which SARS-CoV-2-infected hACE2-A549 cells were used as virus donors for infecting hACE2-RFP-A549 cells. SARS-CoV-2 infection, measured based on NSP3 expression in cocultured hACE2-RFP-A549 cells, was only slightly reduced by NAb treatment (Fig. 1c and d), indicating NAb-resistant cell-to-cell transmission. The infection rate of both cell-free and cell-to-cell transmission was greatly reduced in A549 cells without exogenous hACE2 expression (Fig. 1f), suggesting the involvement of hACE2 in both modes of SARS-CoV-2 transmission. Taken together, our data suggest that SARS-CoV-2 infection proceeds through both cell-free and cell-to-cell modes among cells expressing hACE2.

### hACE2 expression levels regulate SARS-CoV-2 transmission

**Fig. 2:**
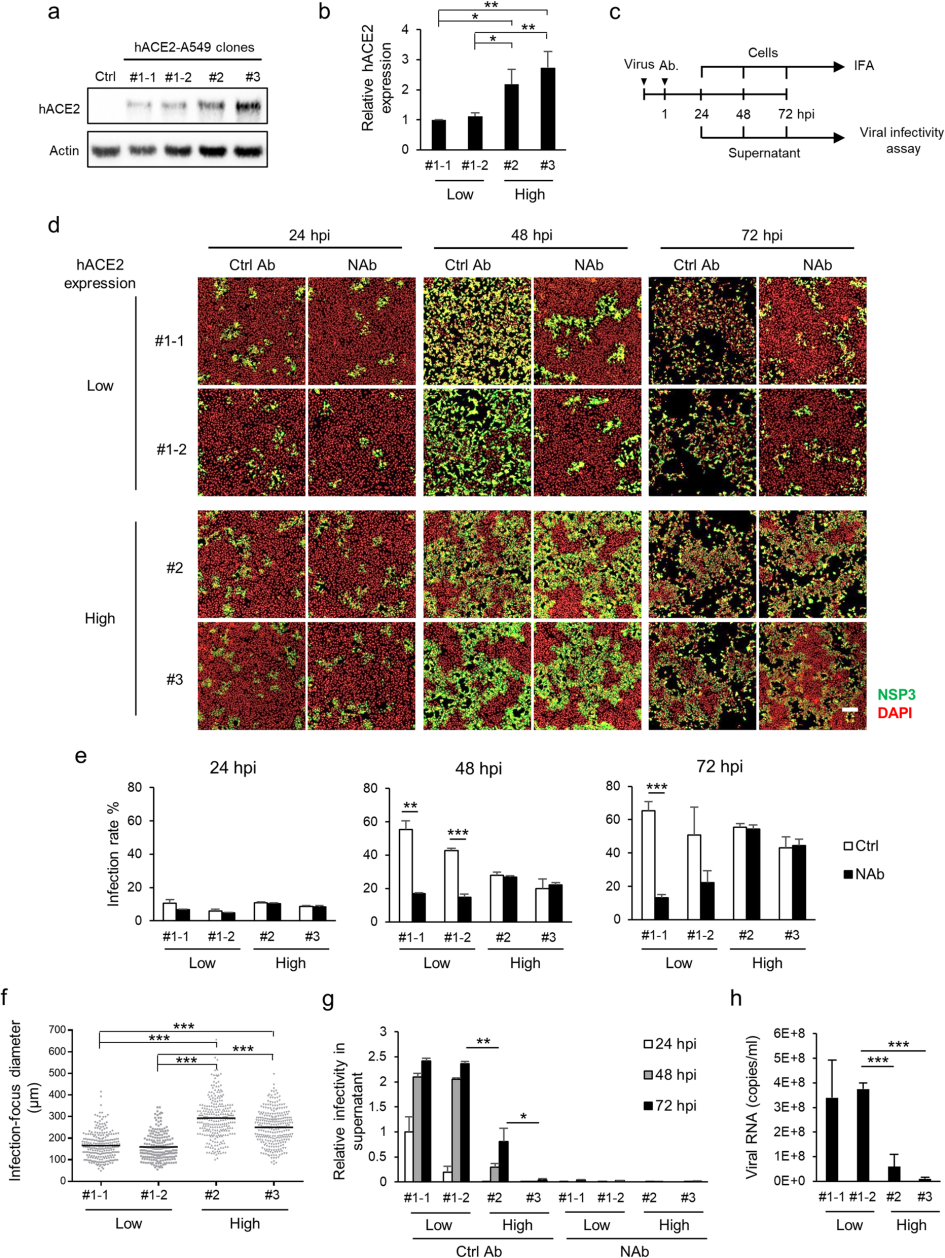
hACE2 expression controls the preference of SARS-CoV-2 infection between cell-free and cell-to-cell transmission. **a** and **b** The hACE2 expression level in each hACE2-A549 clone was determined using Western blotting with an anti-ACE2 antibody, and then quantified utilizing ImageJ software. hACE2 expression was normalized to actin expression, and the #1–1 clone was defined as 1. **c**–**e**, hACE2-A549 clones were adsorbed with SARS-CoV-2 at an MOI of 0.01 for 1 h, and then the cells were replenished with fresh medium containing NAb or Ctrl Ab. At 24, 48, and 72 hpi, the cells were fixed for IFA with anti-NSP3 antibody (**d**), and the virus infection rate was quantified with a high-content image analysis system (**e**). **f** The sizes of infected foci in NAb treatment groups at 48 hpi were measured with over 200 infected foci for each clone. **g** The supernatants at each time point were harvested for virus infectivity assay. The virus infectivity in supernatant from the #1–1 hACE2-A549 clone at 24 hpi was defined as 1. **h** vRNA in supernatant from each hACE2-A549 clone was quantified using real-time RT-PCR at 24 hpi. Low: low hACE2 expression; High: high hACE2 expression; scale bar, 200 μm; data indicated means (**f**) or means with SD (n = 3) (**b**, **e**, **g** and **h**) of each group; *p < 0.05, **p < 0.01, ***p < 0.001, determined using two-tailed unpaired Student’s t test

SARS-CoV-2 infection has been reported to induce dynamic hACE2 expression in humans [10]. To determine whether different hACE2 expression levels affect the mode of transmission, we examined SARS-CoV-2 infection in several A549 cell clones with different expression levels of the hACE2 protein (Fig. 2a and b, and Additional file 2: Fig. S2). To monitor virus progeny transmission over a long period of infection, we infected cells with SARS-CoV-2 at a low MOI in the presence of control antibody or NAb for up to 3 days (Fig. 2c). Notably, different distribution patterns of infected cells were noted between cells with low hACE2 expression (hACE2-A549 #1–1 and #1–2; low-hACE2 cells) and high hACE2 expression (hACE2-A549 #2 and #3; high-hACE2 cells; Fig. 2d).

Compared with control antibody, NAb treatment significantly reduced virus spread in low-hACE2 cells, but not in high-hACE2 cells, at 48 and 72 hpi (Fig. 2d and e). Furthermore, the infection foci in high-hACE2 cells were significantly bigger than those in low-hACE2 cells, especially at 48 hpi (Fig. 2f). The clusters of infected high-hACE2 cells expanded faster than those of infected low-hACE2 cells in the presence of NAb, implying that cell-to-cell transmission was more efficient in high-hACE2 cells. Furthermore, the viral infectivity measured in the culture supernatant indicated that much more infectious SARS-CoV-2 virions were released from low-hACE2 cells than from high-hACE2 cells (Fig. 2g, control antibody), despite a similar level of infected cells (measured based on the expression of the viral NSP3 protein) in both high-hACE2 and low-hACE2 cells (Fig. 2d). To determine the cause for the discrepancy in infectivity, we measured the amount of viral RNA (vRNA) in the supernatant by using real-time RT-PCR. vRNA levels were lower in high-hACE2 cells (Fig. 2h), suggesting that higher hACE2 expression leads to the lower release of progeny virus. Our data indicated that compared with cell-free transmission, cell-to-cell transmission of SARS-CoV-2 is predominant in infected high-hACE2 cells, and the opposite was also true in infected low-hACE2 cells.

### SARS-CoV-2 transmits through intercellular extensions

**Fig. 3:**
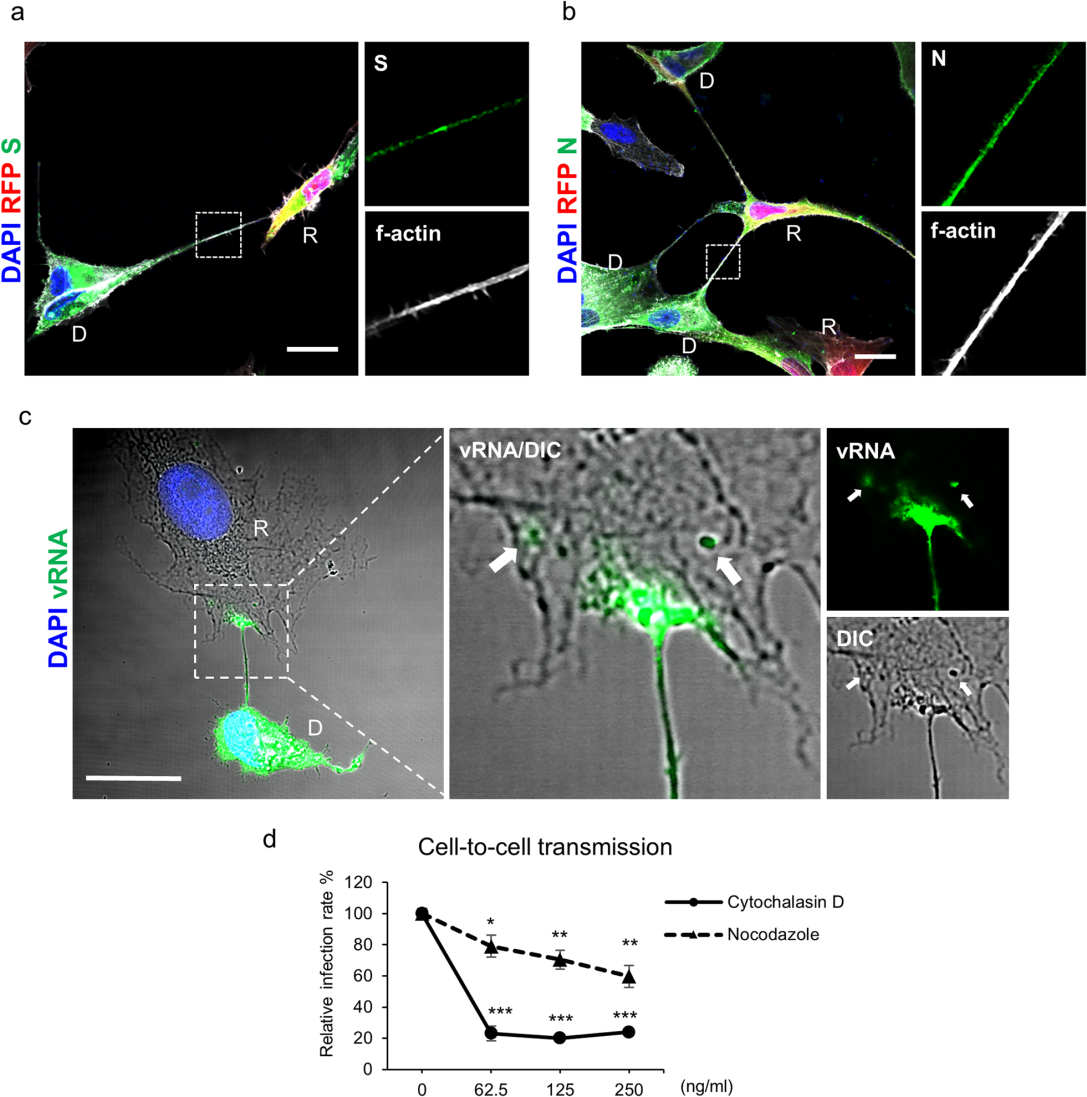
SARS-CoV-2 is transmitted among cells through intercellular extensions. **a–c** The super-resolution immunofluorescence images of cell-to-cell transmission assay at 24 h after coculture. S proteins (**a**) and N proteins (**b**) were stained with antibodies. vRNA (**c**) was detected using a vRNA probe. D: virus donor cells (SARS-CoV-2-infected hACE2-A549 cells); R: hACE2-RFP-A549 cells; arrow: vRNA in recipient cells (**c**); scale bar, 10 μm. **d** Cells were treated with cytoskeleton inhibitors during cell-to-cell transmission assay. The infection of R cells was analyzed using IFA with anti-NSP3 antibody and quantified with a high-content image analysis system. Solvent control groups were defined as 100%; the data indicated means with SD (n = 3) of each group; *p < 0.05, **p < 0.01, ***p < 0.001, determined using two-tailed unpaired Student’s t test

It has been demonstrated that cells can internalize extracellular vesicles through various endocytic pathways [20]. To investigate whether cell-to-cell transmission of SARS-CoV-2 occurs via extracellular vesicles, we introduced endocytosis inhibitors into the experimental setup of cell-to-cell transmission. The findings revealed that the use of endocytosis inhibitors did not lead to a substantial reduction in SARS-CoV-2 cell-to-cell transmission (Additional file 3: Fig. S3). To elucidate the characteristics of cell-to-cell transmission, we employed super-resolution microscopy to examine the subcellular distribution of viral components such as S and N proteins, as well as vRNA. The formation of intercellular extensions connecting virus donor and recipient cells was noted in our coculture system (Fig. 3a–c). Some of the extensions were elongated with a length of up to 100 µm (data not shown), and the morphology was similar to that of tunneling nanotubes [21, 22], which has been reported to be involved in cell-to-cell transmission of SARS-CoV-2 [6]. Furthermore, S proteins (Fig. 3a), N proteins (Fig. 3b), and vRNA (Fig. 3c) were observed in intercellular extensions. vRNA signals in the body of recipient cells were close to the vRNA-containing intercellular extension (Fig. 3c, arrow), suggesting that the virus was transported from the donor cells through intercellular extension to the recipient cells. Thus, SARS-CoV-2 can be transmitted directly through intercellular extensions.

To verify the importance of intercellular extensions in the cell-to-cell transmission of SARS-CoV-2, we treated the coculture system with cytochalasin D and nocodazole for disrupting F-actin and microtubule assembly, which are involved in the formation of intercellular extensions and in cargo transportation, respectively [23]. This treatment significantly reduced cell-to-cell transmission in a nearly dose-dependent manner (Fig. 3d). Thus, our data suggest that SARS-CoV-2 infection spreads directly to neighboring cells through intercellular extensions.

### SARS-CoV-2 virions are packaged in hACE2-containing vesicles for intercellular transportation

**Fig. 4:**
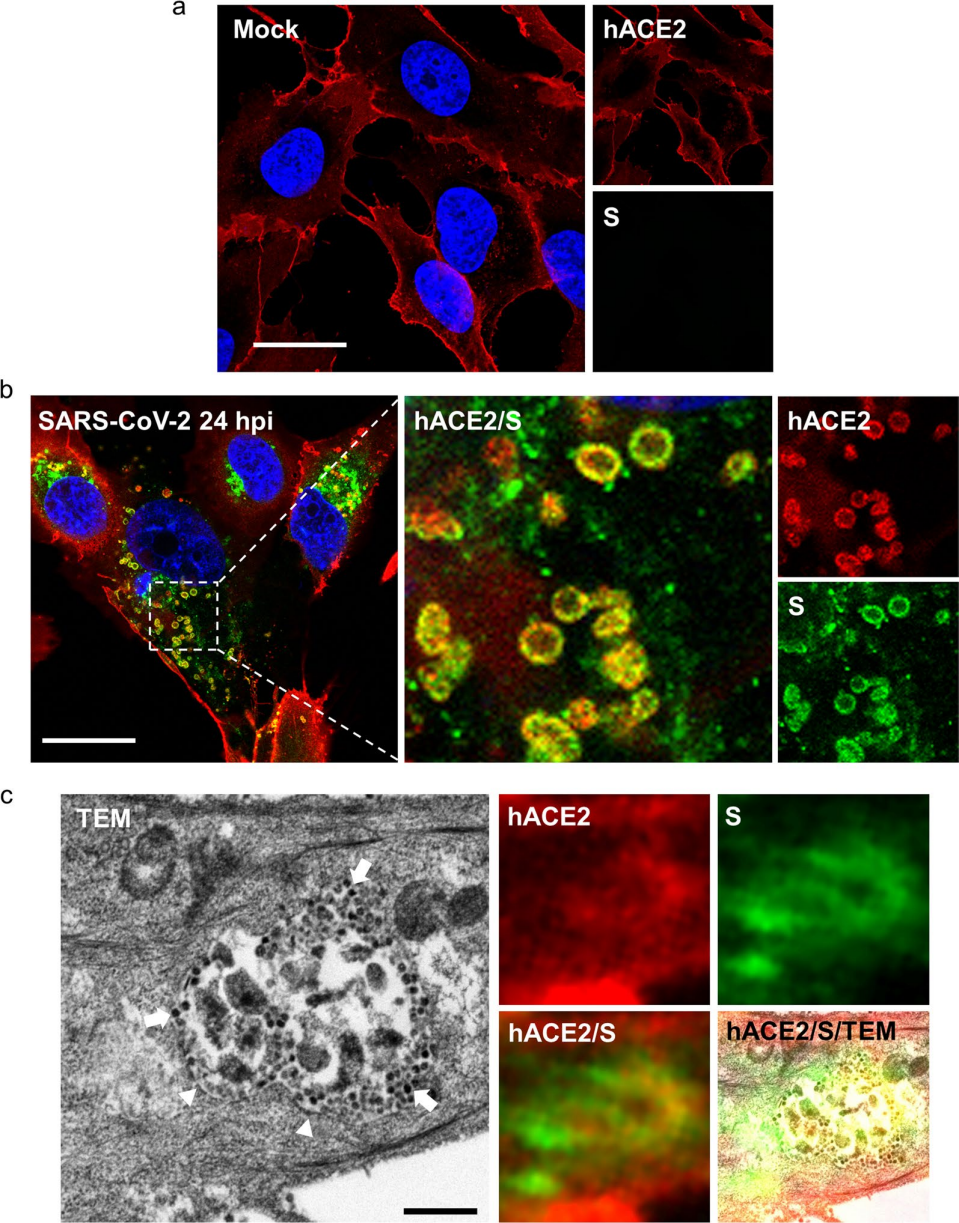
Progenies of SARS-CoV-2 are packaged in hACE2-containing vesicles. Super-resolution immunofluorescence images of mock control (**a**) and SARS-CoV-2-infected hACE2-A549 cells (MOI = 0.2, 24 hpi, **b**). Scale bar, 20 μm (**a** and** b**). **c** CLEM image from SARS-CoV-2-infected hACE2-A549 cells (MOI = 0.2, 24 hpi), which combined the immunofluorescence images and TEM images from the same sample. Arrowhead, single membrane; arrow, VLSs; scale bar, 500 nm

To understand how the viral-entry receptor hACE2 affects SARS-CoV-2 transmission, we monitored the intracellular distribution of hACE2 proteins in infected cells through super-resolution immunofluorescence imaging and TEM. In mock-infected cells, hACE2 proteins were mainly located on the cell surface (Fig. 4a), whereas they were noted around intracellular vesicles and colocalized with viral S proteins in SARS-CoV-2-infected cells at 24 hpi (Fig. 4b). Thus, during SARS-CoV-2 replication, hACE2 proteins were likely redistributed to intracellular vesicles.

To investigate the ultrastructure of hACE2/S-protein-positive vesicles, we used a CLEM approach [24], which enables the acquisition of both immunofluorescence images and TEM micrographs from the same sample. The hACE2/S-protein-positive vesicles appeared to be single-membrane vesicles (SMVs) (Fig. 4c, arrowhead in TEM image) and contained virus-like structures (VLSs) (Fig. 4c, arrow) with a diameter of 80–100 nm, the size of which was similar to the size of SARS-CoV-2 [25, 26]. Notably, these SMVs differed from the double-membrane compartments responsible for SARS-CoV-2 replication [27], suggesting that these are post-replication vesicles. Furthermore, the VLSs appeared to accumulate near the inner surface of the SMV (Fig. 4c), which was similar to the localization of S protein signals in the super-resolution immunofluorescence images (Fig. 4b). Thus, we suspect that these VLSs were SARS-CoV-2 virions produced after virus replication.

**Fig. 5:**
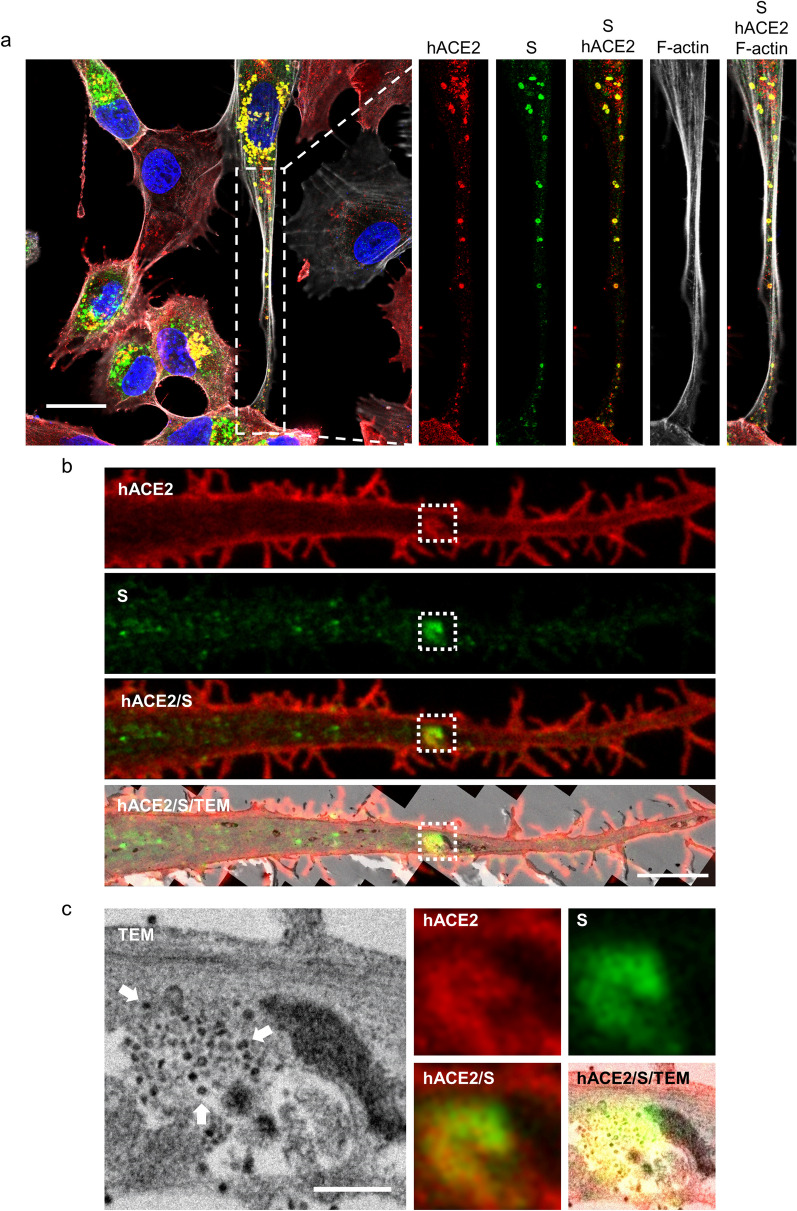
SARS-CoV-2 particles are transported by hACE2-containing vesicles through cellular extensions. **a** Super-resolution immunofluorescence images from SARS-CoV-2-infected hACE2-A549 cells (MOI = 0.2, 24 hpi). Scale bar, 20 μm. **b**, **c** CLEM images from SARS-CoV-2-infected hACE2-A549 cells (MOI = 0.2, 24 hpi). **c** The magnified images from the white dotted frame in **b**. Arrow, VLSs; scale bar, 50 μm (**b**) and 500 nm (**c**)

To determine whether SARS-CoV-2 was transported through intercellular extensions, we examined the super-resolution immunofluorescence images and several CLEM micrographs (Fig. 5). The hACE2/S-protein-positive vesicles were transported along intercellular extensions (Fig. 5a and b), and VLSs were accumulated inside these vesicles (Fig. 5c). These data confirm our finding that the cell-to-cell transmission of SARS-CoV-2 occurs through intercellular extensions and suggest that the hACE2-containing vesicles are likely the vehicles of virus transmission. Overall, our study demonstrated that increased hACE2 expression may cause switching from cell-free transmission to cell-to-cell transmission for SARS-CoV-2 infection, and that hACE2 also plays a role in virus transportation through intercellular extensions.

## Discussion

**Fig. 6:**
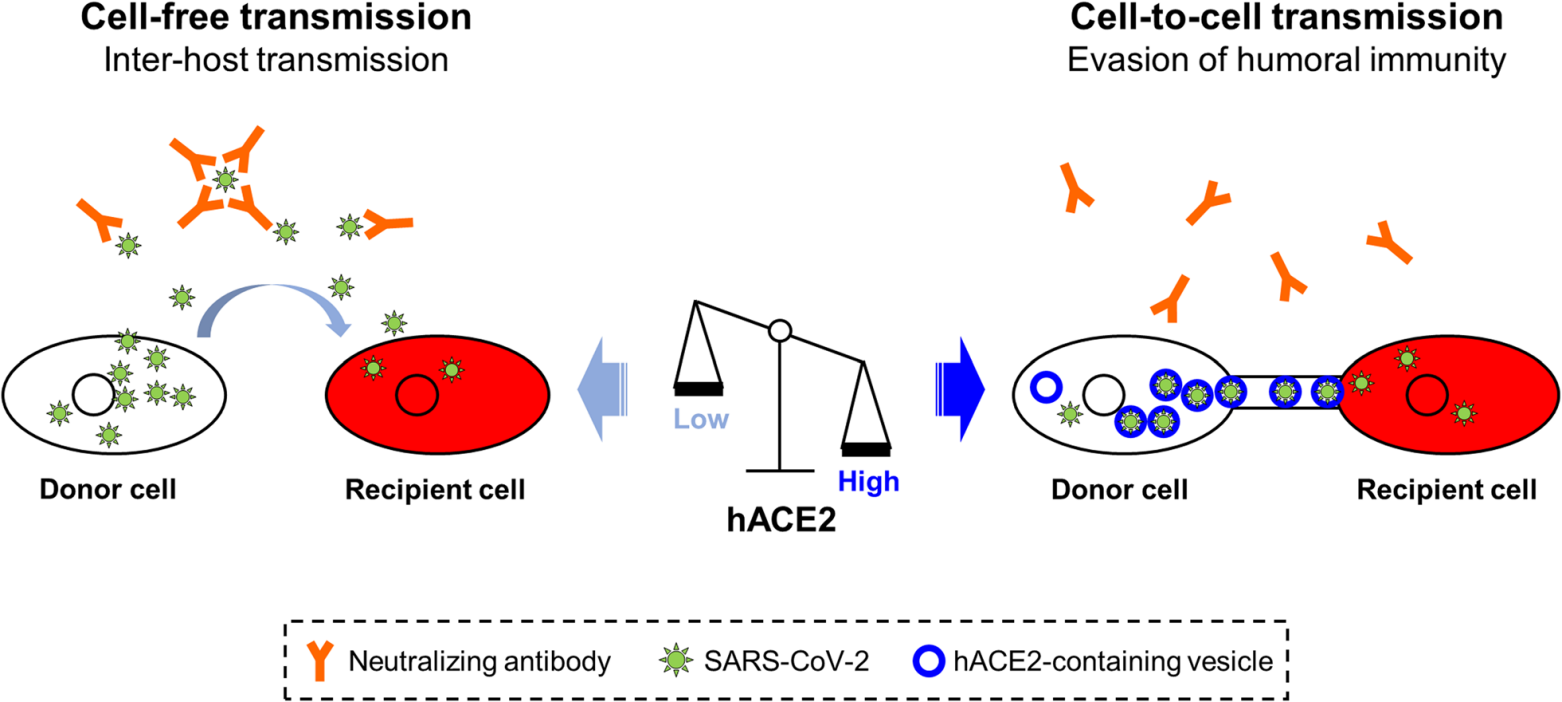
The model of hACE2-regulated SARS-CoV-2 transmission. The hACE2 protein is crucial in regulating the cell-free and cell-to-cell routes of SARS-CoV-2 transmission. When hACE2 levels are limited in cells, the balance tips toward cell-free transmission. Conversely, when hACE2 levels are abundant in cells, the balance tilts toward cell-to-cell transmission

Our results demonstrated that hACE2 is a molecular switch for the mode of SARS-CoV-2 transmission. We propose a model of hACE2-regulated SARS-CoV-2 transmission, in which hACE2 serves as a molecular switch that controls the balance between cell-free and cell-to-cell transmission (Fig. 6). In hACE2 limited cells, the balance tilts toward cell-free transmission, with SARS-CoV-2 infected cells releasing numerous virions, benefiting long-distance viral spread. In hACE2 abundant cells, the balance tilts toward cell-to-cell transmission, with SARS-CoV-2 spreading to neighboring cells likely through hACE2-containing vesicles and intercellular extensions, thus evading humoral immunity.

It is perplexing how the hACE2-limited environment in the lung facilitates high SARS-CoV-2 transmissibility. Between 44 and 69% of SARS-CoV-2 infections are estimated to be transmitted from presymptomatic donors [28, 29], creating major challenges for disease prevention and control. Our finding that SARS-CoV-2 shedding is negatively regulated by hACE2 expression supports the notion of high-level viral shedding in early stages of COVID-19 [28, 30]; this massive release of virions in the low-hACE2 environment in the lung may facilitate interhost transmission. Additionally, the early peak of SARS-CoV-2 shedding preceding the induction of antibody-mediated immune responses [28, 30, 31] could further increase the likelihood of virus spread.

Similar phenomena of decreased viral shedding in an environment with high levels of viral receptors have been reported for other viruses. For instance, sialic acids, the receptors for influenza virus, capture budding progeny virions on the cell surface and inhibit their release while viral neuraminidase is inactivated [32]. The suppression of viral shedding by neuraminidase inhibitors remains the primary therapeutic strategy against flu [33], emphasizing the importance of receptor–virus interactions in viral shedding and pathogenesis. In the present study, we observed that many SARS-CoV-2 particles accumulated in hACE2-containing SMVs. This virion accumulation may be due to the abundant hACE2 proteins on the inner surface of SMVs, which retain the virions through a mechanism similar to the sialic acid–influenza virus interaction. These hACE2-containing vesicles likely transport progeny SARS-CoV-2 particles for cell-to-cell transmission through intercellular extensions. Notably, our observation that SARS-CoV-2 particles were inside the intercellular extensions making them more likely to evade NAb attacks, compared with the virions observed on (outside) intercellular extensions in previous studies [6, 7]. However, whether the SARS-CoV-2 accumulation in hACE2-containing vesicles is the cause or the consequence of the switch to cell-to-cell transmission requires further study.

We also demonstrated that increased hACE2 expression promotes the cell-to-cell transmission of SARS-CoV-2. *ACE2* is an ISG that is upregulated following viral infection in humans after the induction of innate immunity and then adaptive immunity. Thus, it is beneficial for viral spread in cases where SARS-CoV-2 infection can switch from cell-free transmission to cell-to-cell transmission to avoid attacks from NAb. Additionally, SARS-CoV-2 may be capable of infecting neurons through cell-to-cell transmission [6], thus potentially contributing to the neurological symptoms associated with COVID-19, including long COVID-19 [34]. However, the exact mechanisms of neuronal infection and the long-term consequences of SARS-CoV-2 infection for neurons remain unclear.

## Conclusions

Our study demonstrated that hACE2 not only functions as the receptor for SARS-CoV-2, enabling viral entry, but also serves as a molecular switch that controls the mode of SARS-CoV-2 transmission. Cell-free transmission and cell-to-cell transmission have different advantages and disadvantages. Their dynamic regulation through hACE2 allows SARS-CoV-2 to maximize its transmission fitness and adapt to different stages of COVID-19 progression in an individual. Furthermore, it is worth noting that neutralizing antibodies can only impede cell-free transmission, while our study demonstrates that hACE2 plays a role in both cell-free and cell-to-cell transmission. Therefore, the use of ACE2 inhibitor in combination with other antivirals may enhance the treatment’s efficacy by halting SARS-CoV-2 transmission. Our study provides new insights into the COVID-19 pandemic in terms of the infectivity and spread of SARS-CoV-2, and the study results can guide the development of new antiviral measures.

### Supplementary Information

**Additional file 1: Figure S1.** SARS-CoV-2 efficiently spreads among hACE2-expressing cells when cell-free transmission is completely blocked by convalescent plasma. Cell-free infection: SARS-CoV-2 was preincubated with convalescent (CP) or control (Ctrl) plasma and then used to infect hACE2-RFP-A549 cells (R) for 24 h. Coculture system: SARS-CoV-2-infected hACE2-A549 cells (MOI = 0.2, 24 hpi) as virus donor cells (D) were cocultured with hACE2-RFP-A549 cells with CP or Ctrl plasma for 24 h. The infection of R cells was determined using IFA with anti-NSP3 antibody (**a**) and quantified with a high-content image analysis system (**b**). The supernatant was harvested for the virus infectivity assay (**c**). Arrowhead, SARS-CoV-2 infected R cells; Scale bar = 50 μm; Ctrl groups were defined as 100%; Data indicated means with standard deviation (SD) (n = 3) of each group.

**Additional file 2: Figure S2.** hACE2 expression level of hACE2-A549 clones. hACE2 expression level of each hACE2-A549 clone was analyzed using Western blotting with anti-ACE2 antibody.

**Additional file 3: Figure S3.** The effect of endocytosis inhibitors on SARS-CoV-2 cell-to-cell transmission. Cells were treated with endocytosis inhibitor (dynasore) or clathrin-mediated endocytosis inhibitor (piststop 2) during cell-to-cell transmission assay. The infection of R cells was analyzed using IFA with anti-NSP3 antibody and quantified with a high-content image analysis system. Cell viability is determined by the total cell count, with nuclei stained using DAPI. Cell counting was performed using a high-content image analysis system. Solvent control groups were defined as 100%; the data indicated means with SD (n = 3) of each group.

## Data Availability

All the data was included in the manuscript and additional file. All the materials and reagent sources used in this study are described in the methods section.

## Abbreviations

hACE2: Human angiotensin-converting enzyme 2
NAb: Neutralizing antibody
S: Viral spike glycoprotein
RBD: Receptor binding domain
N: Viral nucleocapsid protein
NSP3: Viral nonstructural protein 3
vRNA: Viral RNA
ISG: Interferon-stimulated gene
SMV: Single-membrane vesicle
VLS: Virus-like structure
IFA: Immunofluorescence assay
MOI: Multiplicity of infection
CLEM: Correlative light and electron microscopy
RT-PCR: Reverse transcription–polymerase chain reaction
TEM: Transmission electron microscopy
hmAb: Humanized monoclonal antibody
hpi: Hours post infection
