# Supplementary figures and images for: Human ACE2 protein is a molecular switch controlling the mode of SARS-CoV-2 transmission
Source: J Biomed Sci. 2023 Oct 12;30:87. doi: 10.1186/s12929-023-00980-w (PMC10571257; doi:10.1186/s12929-023-00980-w)

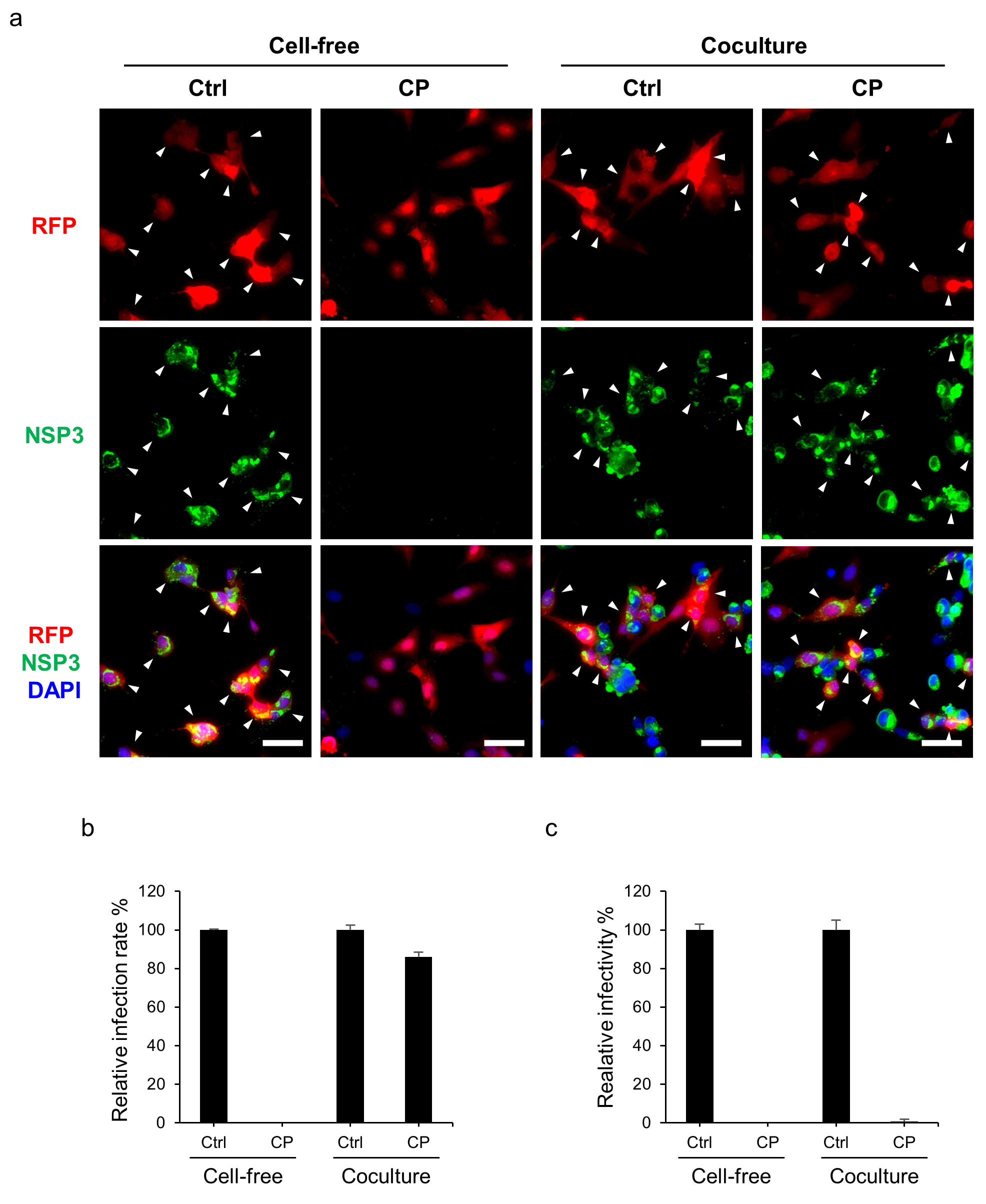

Supplement: Supplementary file 1 — Additional file 1: Figure S1. SARS-CoV-2 efficiently spreads among hACE2-expressing cells when cell-free transmission is completely blocked by convalescent plasma. Cell-free infection: SARS-CoV-2 was preincubated with convalescent (CP) or control (Ctrl) plasma and then used to infect hACE2-RFP-A549 cells (R) for 24 h. Coculture system: SARS-CoV-2-infected hACE2-A549 cells (MOI = 0.2, 24 hpi) as virus donor cells (D) were cocultured with hACE2-RFP-A549 cells with CP or Ctrl plasma for 24 h. The infection of R cells was determined using IFA with anti-NSP3 antibody (a) and quantified with a high-content image analysis system (b). The supernatant was harvested for the virus infectivity assay (c). Arrowhead, SARS-CoV-2 infected R cells; Scale bar = 50 μm; Ctrl groups were defined as 100%; Data indicated means with standard deviation (SD) (n = 3) of each group. [file 12929_2023_980_MOESM1_ESM.tif]

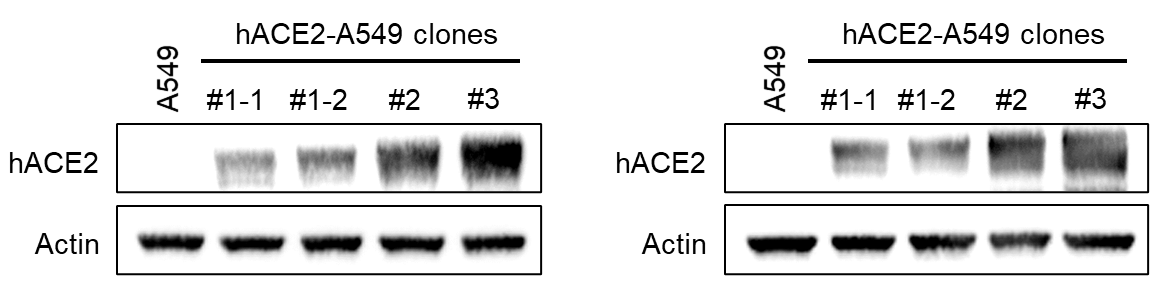

Supplement: Supplementary file 2 — Additional file 2: Figure S2. hACE2 expression level of hACE2-A549 clones. hACE2 expression level of each hACE2-A549 clone was analyzed using Western blotting with anti-ACE2 antibody. [file 12929_2023_980_MOESM2_ESM.tif]

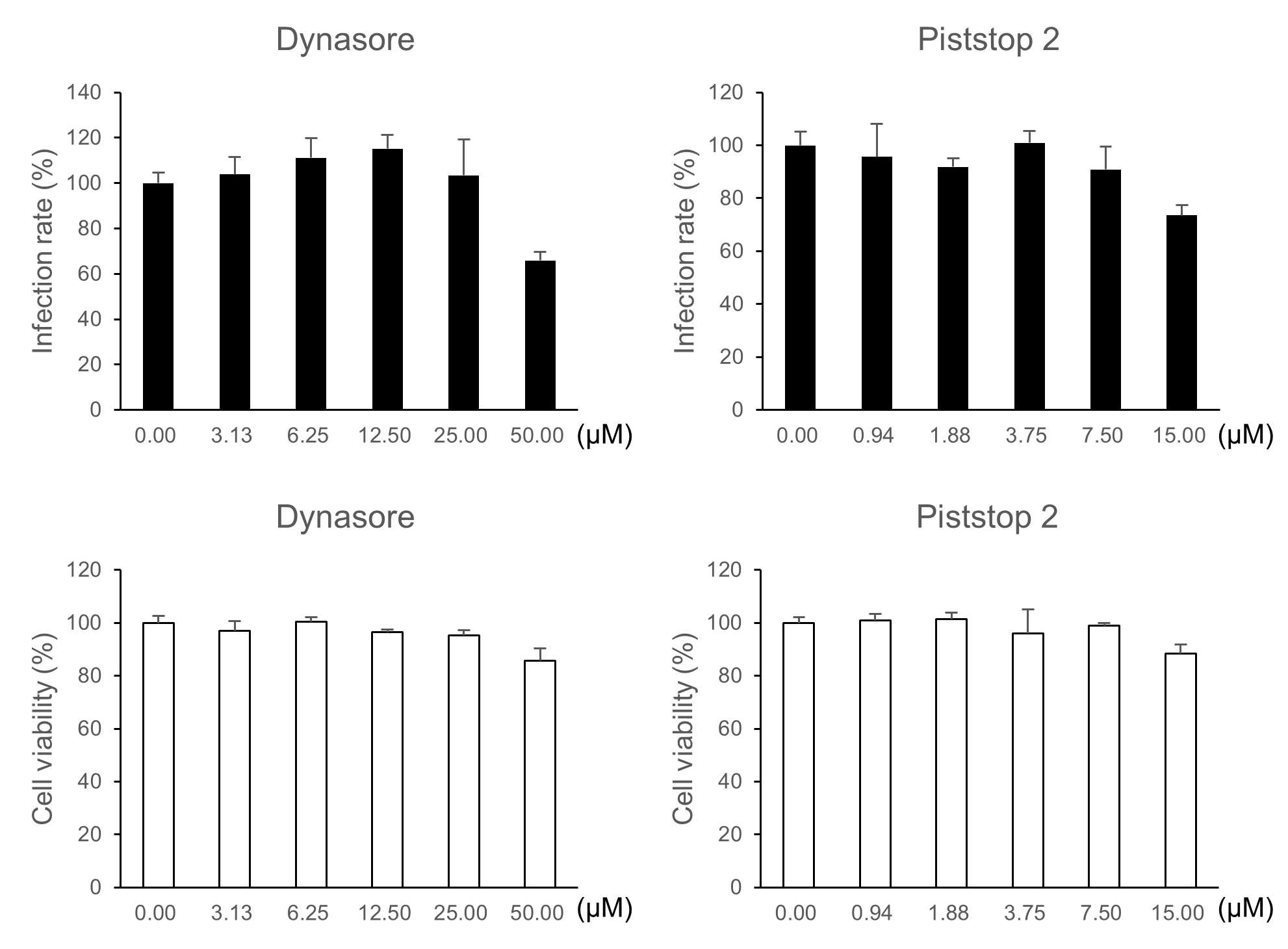

Supplement: Supplementary file 3 — Additional file 3: Figure S3. The effect of endocytosis inhibitors on SARS-CoV-2 cell-to-cell transmission. Cells were treated with endocytosis inhibitor (dynasore) or clathrin-mediated endocytosis inhibitor (piststop 2) during cell-to-cell transmission assay. The infection of R cells was analyzed using IFA with anti-NSP3 antibody and quantified with a high-content image analysis system. Cell viability is determined by the total cell count, with nuclei stained using DAPI. Cell counting was performed using a high-content image analysis system. Solvent control groups were defined as 100%; the data indicated means with SD (n = 3) of each group. [file 12929_2023_980_MOESM3_ESM.tif]
